# Supplementary material for: Carotid intima-media thickness in polycystic ovary syndrome and its association with hormone and lipid profiles
Source: PLoS One. 2020 Apr 24;15(4):e0232299. doi: 10.1371/journal.pone.0232299 (PMC7182264; doi:10.1371/journal.pone.0232299)
Supplement: Attachment — Submitted filename: Response_To_Reviewers_Rhea_Jabbour.docx [file pone.0232299.s002.docx]

Medical University of Vienna, Department of Obstetrics and Gynecology

Division of Gynecological Endocrinology and Reproductive Medicine

Währinger Gürtel 18-20, A-1090 Vienna, Austria, Tel: +43-1-40400 28160, Fax: +43-1-40400 28170

**Rhea Jabbour**, M.D. c/o **Peter Frigo**, M.D., Ph.D	Email: [rhea.jab@gmail.com](mailto:rhea.jab@gmail.com)	Tel: +43 676 44 09 446

**Rebuttal Letter**

Please find below our response to the points raised by the reviewers. The mentioned lines of the manuscript correspond to those of the document “Revised_Manuscript_With_Track_Changes_Rhea_Jabbour”, as they differ from the ones in the Manuscript without tracked changes.

**Reviewer #1:**

**The authors have addressed all the comments of the reviewers and the manuscript has been improved. However, I have additional minor comments:**

**-In the sentence (592-599): “Possible explanations could be a protective effect of hyperandrogenism, especially DHEAS, in menopause, or delayed menopause in PCOS.” I disagree with this statement and I find it contradictory to what is stated in the article. It seems to me that it is unlikely that hyperandrogenism (DHEAS) has a protective effect. Probably to be an estrogenic effect, as some articles show. “Meun et al. hypothesize that later menopause with prolonged estrogen exposure may have a protective role against CVD. (Meun, C., J Clin Endocrinol Metab. 2018; 103: 1622–1630).**

**Although some articles have reported that PCOS women have lower basal estradiol level, it has also been described that this value would be comparable to control women. Moreover, a study has showed higher peak estradiol level in PCOS patients (Eur J Obstet Gynecol Reprod Biol. 2013 Sep;170(1):165-70). Which could favor the testosterone/estradiol ratio towards estradiol effect as women with PCOS are older and androgens decreased.**

Reply: Thank you very much for your valuable comment. We have clarified this point as exposed below.

l.388-396: “However, although women with PCOS feature increased CIMT and metabolic and cardiovascular risk at a younger age, there is evidence suggesting that they do not show higher prevalence of cardiovascular events than controls after menopause[30,64,65]. Possible explanations could be a protective effect of delayed menopause with a consequently prolonged estrogen exposure in PCOS[30,64,65] or even hyperandrogenism itself[64,65] in peri- and postmenopausal PCOS patients, as suggested by previous studies[27,28,29], probably mainly due to enzymatic conversion to estrogen. Moreover, differences in cardiovascular risk factors (e.g. diabetes, abdominal obesity) between PCOS and controls seem to be less preponderant in aging women, explaining the similar cardiovascular morbidity and mortality later in life[65].”

**-The range of BMI that the authors report is quite wide (17-50 kg/m2). Atypical outcomes are usually observed in women with BMI> 40. Did you observe any particularity in them or behave within the average? Remember the contribution of steroids in the adipose tissue of women with high BMI.**

Reply: We have only included 4 PCOS patients and no controls with a BMI > 40 kg/m^2^ in our study. Therefore, the sample size was too small in order to be able to analyze any atypical outcomes in these subjects.

**-Regarding transvaginal ultrasound that was performed in 6 regularly-cycling women in order to exclude PCOS phenotype. How long after the initial study was performed? Remember that the ovarian image may vary over time. I suggest indicating in materials and methods so that the interpretation is at the reader criteria**

Reply: Thank you very much for your suggestion. In fact, transvaginal ultrasound was performed either on the same day of blood tests, if past medical history suggested hyperandrogenemia, or the following day, once laboratory results were obtained. It was therefore performed between the third and the fifth day of the subjects’ menstrual cycle. We have included this precision in the Materials and Methods l.121-123:

l.121-123: “PCOS was ruled out in all controls according to the Rotterdam ESHRE/ASRM criteria[3], as transvaginal ultrasound was performed between the third and the fifth day of their menstrual cycle in case of hyperandrogenism to exclude PCOS phenotype 3.”

**Reviewer #2:**

**The authors revised the manuscript generally well according to my comments.
However, I still have a question. Have the authors analyzed if there is a difference in CIMT between obese PCOS patients and non-obese PCOS patients? It is recommended to present the results of this subgroup analysis.**

Reply: Thank you very much for your feedback. Our sample size was too small in order to be able to compare CIMT between obese and non-obese PCOS patients. A larger sample size would be needed in order to yield statistical significance, as described in the Discussion l.418-420.

However, the impact of obesity on CIMT was analyzed in this study among the entire study population. Our results showed that visceral obesity was positively associated with CIMT, both in correlation analysis (see l.258-259) and multiple linear regression analysis (see l.274-275), as BMI represented an independent positive predictor of CIMT according to model 2. However, as stated in l.276-278, “PCOS status remained the primary predictor of CIMT, even after multiple adjustments for BMI, age and smoking status (*P*<0.001, R^2^=0.73, models 3 and 4), therefore indicating an independent effect of PCOS on CIMT (β=0.797, *P*<0.001)”.

**Reviewer #3:**

**The Authors clarified different points of the analysis improving the overall value of the manuscript that can be considered for publication.**

Reply: Thank you very much for your feedback and your positive recommendation.
